# Integration of digital dental casts in cone beam computed tomography scans—a clinical validation study

**DOI:** 10.1007/s00784-017-2203-2

**Published:** 2017-09-20

**Authors:** Frits A. Rangel, Thomas J. J. Maal, Martien J. J. de Koning, Ewald M. Bronkhorst, Stefaan J. Bergé, Anne Marie Kuijpers-Jagtman

**Affiliations:** 1 Department of Orthodontics and Craniofacial Biology, Radboud University Medical Centre, 309 Dentistry, PO Box 9101, 6500 HB Nijmegen, The Netherlands; 2 Department of Oral and Maxillofacial Surgery, Radboud University Medical Centre, Nijmegen, The Netherlands; 3 Department of Preventive and Restorative Dentistry, Radboud University Medical Centre, Nijmegen, The Netherlands

**Keywords:** Orthodontics, Oral and maxillofacial surgery, Imaging, Three-dimensional, Three-dimensional imaging, Computer-assisted, Digital dental casts

## Abstract

**Objectives:**

Images derived from cone beam computed tomography (CBCT) scans lack detailed information on the dentition and interocclusal relationships needed for proper surgical planning and production of surgical splints. To get a proper representation of the dentition, integration of a digital dental model into the CBCT scan is necessary. The aim of this study was to validate a simplified protocol to integrate digital dental models into CBCT scans using only one scan.

**Materials and methods:**

Conventional protocol A used one combined upper and lower impression and two CBCT scans. The new protocol B included placement of ten markers on the gingiva, one CBCT scan, and two separate impressions of the upper and lower dentition. Twenty consecutive patients, scheduled for mandibular advancement surgery, were included. To validate protocol B, 3-dimensional reconstructions were made, which were compared by calculating the mean intersurface distances obtained with both protocols.

**Results:**

The mean distance for all patients for the upper jaw is 0.39 mm and for the lower jaw is 0.30 mm. For ten out of 20 patients, all distances were less than 1 mm. For the other ten patients, all distances were less than 2 mm.

**Conclusions:**

Mean distances of 0.39 and 0.30 mm are clinically acceptable and comparable to other studies; therefore, this new protocol is clinically accurate.

**Clinical relevance:**

This new protocol seems to be clinically accurate. It is less time consuming, gives less radiation exposure for the patient, and has a lower risk for positional errors of the impressions compared to other integration protocols.

## Introduction

In maxillofacial imaging, creating three-dimensional (3D) digital datasets has become the standard. Now that the integration of different datasets is established [1–3], treatment planning in maxillofacial surgery has shifted to a digital 3D approach. Using 3D digital datasets, treatment can be planned in advance, making surgery more predictable and reducing the time in the operation theater [4, 5]. Most researchers and clinicians agree that images derived from cone beam computed tomography (CBCT) scans do not provide enough detailed information about the dentition and interocclusal relationships for treatment planning purposes. This is because of the limited scanning resolution and streak artifacts caused by radiopaque dental restorations or orthodontic brackets [3, 6–11]. Consequently, to obtain a proper representation of the dentition, integration of a digital dental model into the CBCT scan is necessary.

Using a surface matching method, the digital dental model can be integrated into the CBCT scan. Especially by using an open mouth posture, small voxel size, and specific segmentation threshold selection, the quality of the integration model is good [12, 13]. However, when patients have metal restorations or orthodontic appliances, surface matching of a digital dental cast onto the dentition in the CBCT scan is nearly impossible. Utilizing intra-oral reference devices or bite jigs to locate fiducial markers outside the occlusal area improves the integration of digital dental casts into CBCT scans [9, 10, 14–18]. This method is adequate; however, it is time consuming and expensive. Customized intra-oral reference devices and bite jigs must be fabricated, and an extra appointment is needed, which is inconvenient to the patient. Besides that, most of the fiducial markers are placed outside the mouth, and support structures run through the lip commissure, resulting in soft tissue distortion. This inhibits a reliable judgment of the soft tissues at rest.

Swennen et al. [3] used a triple scan method to integrate a high-resolution 3D image of the dentition into the CBCT scan. Their method is reliable and does not cause any soft tissue deformation. The disadvantage, however, is that two CBCT scans are needed for the integration model. Although the second CBCT scan is a so-called low-dose scan, it contributes to the stochastic effect of radiation exposure. Therefore, a method that produces the same result without the need for the second scan is preferable. Rangel et al. [8] introduced a method where titanium markers were glued on the gingiva, which were then used for the matching procedure. Using this method, only one CBCT is needed. The aim of this study is to evaluate digital planning when using this new method introduced by Rangel et al. [8] compared to the Swennen protocol [3].

## Materials and methods

### Study sample

From the department of Orthodontics and Craniofacial Biology of the Radboud University Nijmegen Medical Centre, 20 consecutive patients were included who were scheduled for mandibular advancement surgery using a bilateral sagittal split osteotomy according to Hunsuck’s modification. Inclusion criteria were defined as follows: (1) healthy patient scheduled for a combined orthodontic-orthognathic surgical treatment, (2) retrognathic mandible, (3) at least 12 teeth present in both the upper and lower dental arch, (4) absence of crowns and prosthetic veneers, (5) all third molars removed at least 6 months before surgery, and (6) an informed consent by the patient. This research was conducted in accordance with the Helsinki Declaration with regard to research in human subjects. Approval from the Institutional Review Board of the Radboud University Medical Centre was requested, and the Board confirmed that ethical approval was not needed (ref. no. 2011/173).

### Imaging protocol

**Fig. 1:**
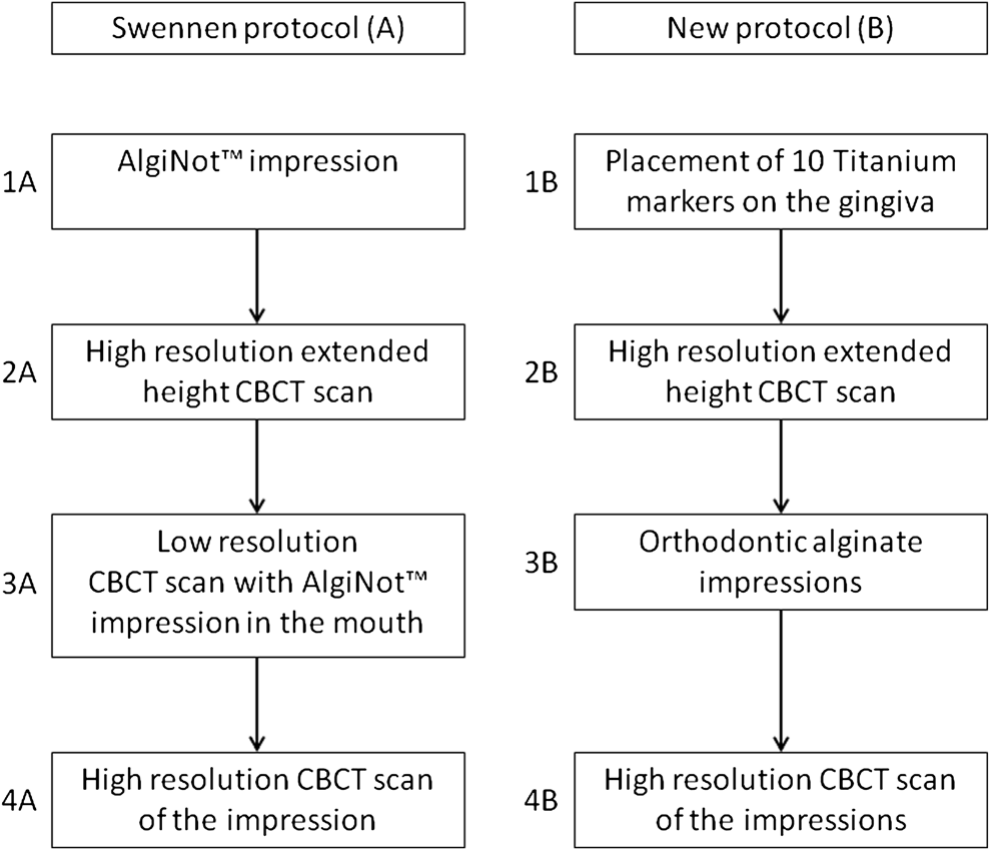
Flowchart showing the four steps for collecting all of the patient data for imaging protocols A and B

The first imaging protocol (A) that was followed was the triple scan method according to Swennen [3]. According to this protocol, four steps were followed (Fig. 1):

1. An impression (AlgiNot™, Kerr USA, Romulus, MI) was made of the upper and lower dentition together.
2. An extended height scan (I-CAT™, Imaging Sciences International, Inc., Hatfield, USA, field of view: 17 cm diameter, 22 cm height; scan time 2 × 20 s; voxel size 0.4 mm) at 120 kV and 47.74 mA was made of the patient in maximal occlusion.
3. The AlgiNot™ impression was placed in the patient’s mouth in the correct position, and a low resolution scan (I-CAT™, Imaging Sciences International, Inc., Hatfield, USA, field of view: 17 cm diameter, 8 cm height; scan time 1 × 10 s; voxel size 0.4 mm) was made at 120 kV and 47.74 mA.
4. The AlgiNot™ impression was scanned (I-CAT™, Imaging Sciences International, Inc., Hatfield, USA, field of view: 17 cm diameter, 13 cm height; scan time 40 s; voxel size 0.2 mm) at 120 kV and 47.74 mA.

The new, second scanning protocol (B) also consisted of four steps (Fig. 1):

1. Five titanium markers (Speed split stops, Strite Industries Limited, Cambridge, Ontario, Canada) were glued (Indermil®, Henkel Ireland Ltd., Whitestown, Dublin, Republic of Ireland) to the gingiva in each jaw.
2. An extended height CBCT scan was made of the patient in maximal occlusion (I-CAT™, Imaging Sciences International, Inc., Hatfield, USA, field of view: 17 cm diameter, 22 cm height; scan time 2 × 20 s; voxel size 0.4 mm) at 120 kV and 47.74 mA.
3. Orthodontic impressions were made using plastic impression trays (TP Orthodontics, Inc., La Porte, Indiana, USA) and orthodontic alginate (Cavex Orthotrace, Cavex Holland BV, Haarlem, The Netherlands). The titanium markers stayed embedded in the alginate when the impression tray was removed.
4. The orthodontic impressions were scanned (I-CAT™, Imaging Sciences International, Inc., Hatfield, USA, field of view: 17 cm diameter, 13 cm height; scan time 40 s; voxel size 0.2 mm) at 120 kV and 47.74 mA.

**Fig. 2:**
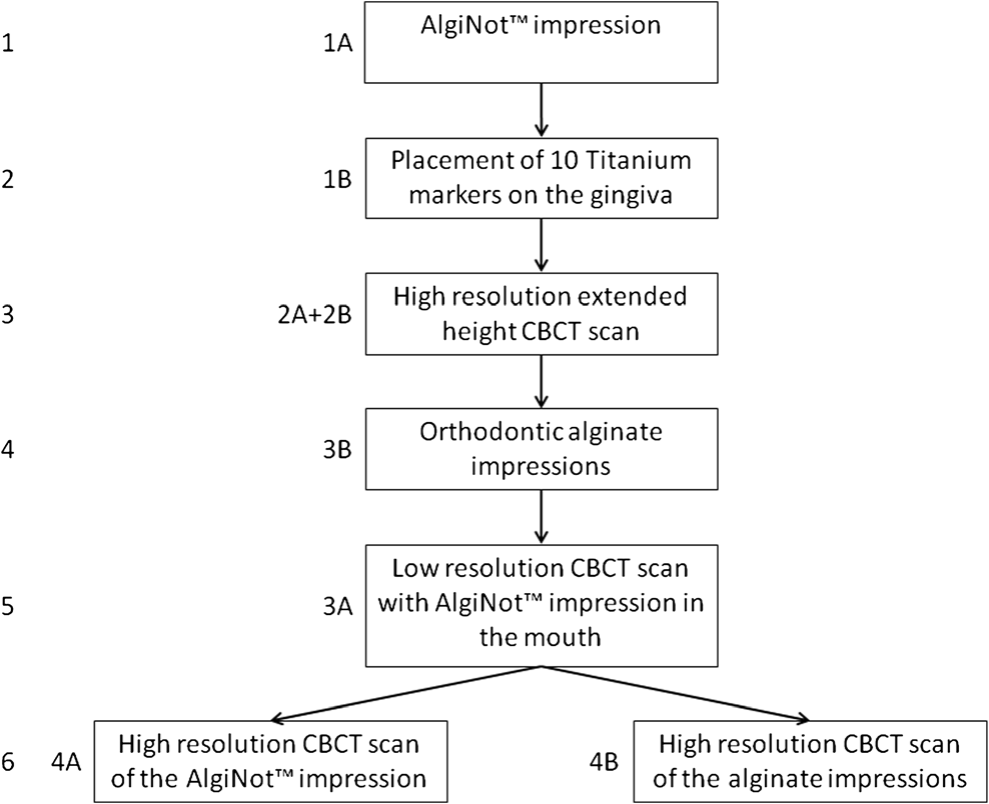
Six-step procedure for gaining all the patient data

To minimize the radiation dose and discomfort to the patient, for the data collection, the two protocols were combined to a six-step procedure (Fig. 2).

### Matching procedure

**Fig. 3:**
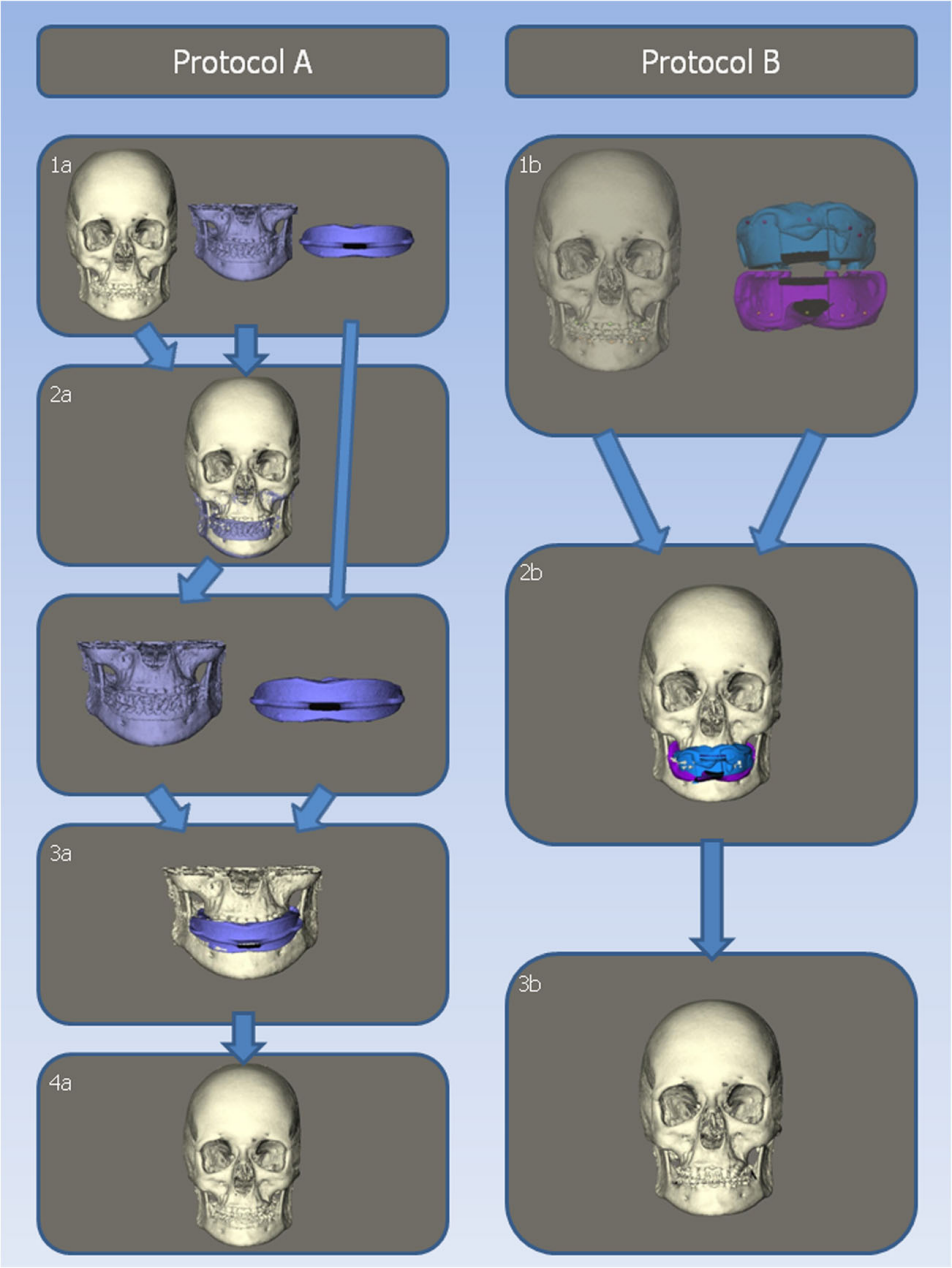
Matching procedure steps for protocols A and B

The acquired data from the CBCT scans were exported in Digital Imaging and Communications in Medicine (DICOM) format to Maxilim 2.3.0.3 (Medicim NV, Mechelen, Belgium). In Maxilim, 3D reconstructions were made for both scanning protocols. Both protocols are illustrated in Fig. 3.

The 3D reconstruction process for protocol A (Swennen) consisted of four steps; this has been described in detail by Swennen et al. 2009 [3]:

- All DICOM files were imported into Maxilim and 3D reconstructions were made.
- The 3D reconstruction of the low resolution scan (3A) was matched on the patient’s skull (2A) using a voxel-based matching procedure.
- The high-resolution impression scan (4A) was matched on the low resolution scan (3A) using a voxel-based matching procedure.
- Out of the impression, a positive image of the teeth was made.

For protocol B (Rangel), the 3D reconstruction consisted of three steps. For a detailed description, see Rangel et al. [8]. In short, the procedure was as follows:

1. 3D reconstruction of the skull of the patient and the impressions out of the extended height scan, with separate extraction of the markers (to get both protocols into the same dataset and in the same coordinate system), the same reconstruction of the skull of the patient was used as for step 1 in protocol A),
2. Marker-based registration of the impressions in the CBCT scan. The centroid of the markers was extracted from both scans to perform the registration [19].
3. Creating a positive image of the teeth out of the impressions.

**Fig. 4:**
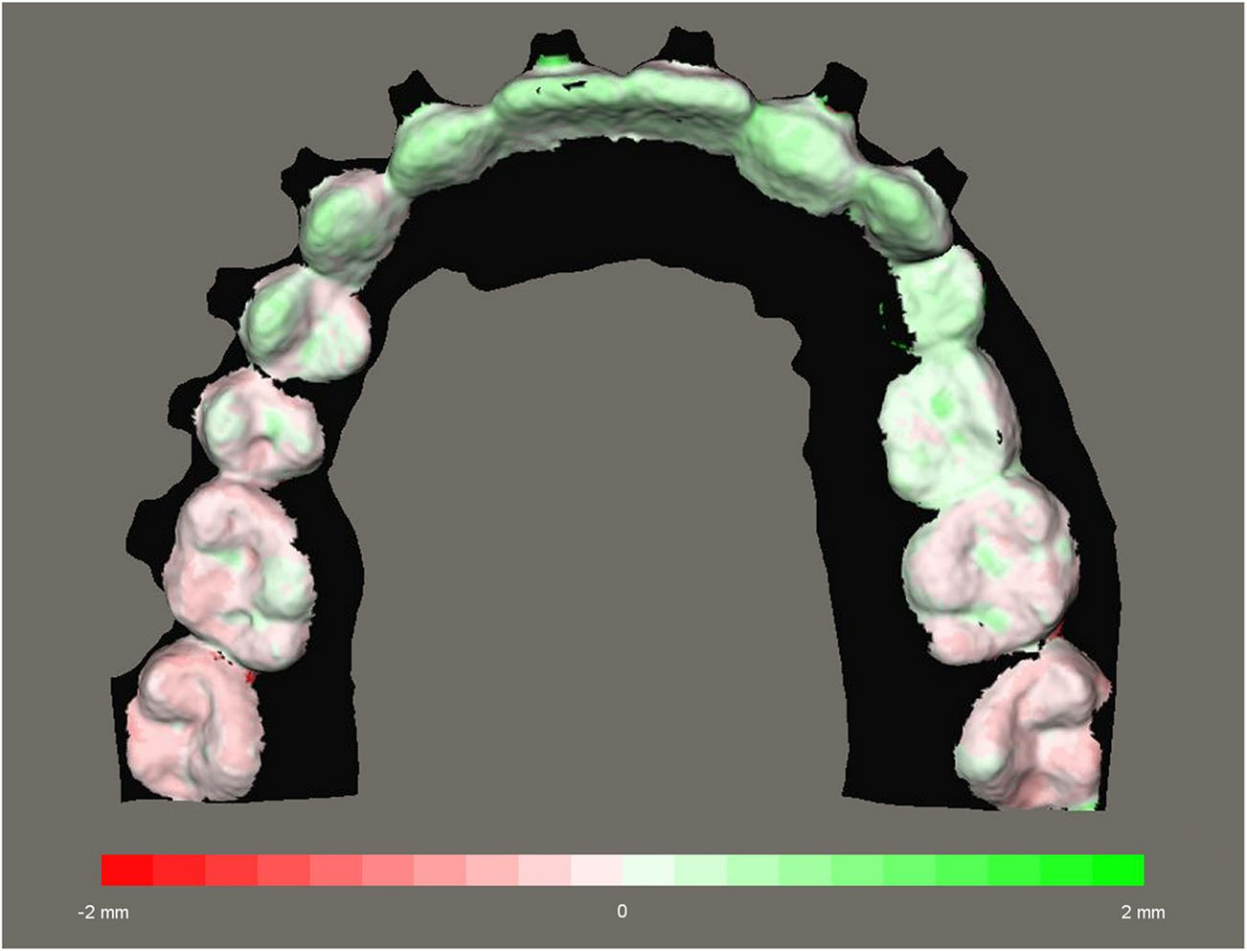
Distance map of the matched dentition using protocols A and B. Values range from − 2.0 to 2.0 mm

The 3D datasets consist of thousands of polygons that are connected to create the dataset. The Maxilim software was used to calculate the distance between corresponding polygons of the two datasets. For this purpose, the dentitions were selected on the final datasets of protocol A (Swennen) and B (Rangel). The area of the brackets was excluded since this part is subject to distortion. Out of these distances, a so-called distance map (Fig. 4) was constructed, visualizing the difference between the two protocols.

### Statistics

The matching process is performed automatically by the software which was previously tested in other studies that is reproducible [19, 20]; therefore, it is reasonable to accept that the matchings will be the same, every time you let the software perform these matchings.

For the upper and lower arches, separate distance maps were made to compare protocols A (Swennen) and B (Rangel). Out of these distance maps, the absolute mean distance, standard deviations, and the 95th percentile were calculated. The 95th percentile means that 95% of all distances are less than that value. Also, the percentage of measurements that are larger than 1 and 1½ mm was calculated.

## Results

### Sample

Twenty patients were included in the study (12 males, 8 females). The mean age of the patients was 27.0 years (SD 11.1 years).

### Intersurface distances

For the upper and lower arches, separate distance kits were made to compare protocols A (Swennen) and B (Rangel). The results for the upper and lower jaw are shown in Tables 1 and 2, respectively. The mean distance between the distance kits is on average 0.39 mm for the upper jaw and 0.30 mm for the lower jaw. In ten of the 20 patients, all measurements in the distance map were less than 1 mm. For the other ten patients, all distances were less than 2 mm. Patient 17 showed the poorest results. For this patient, the 95th percentile is 1.86 mm for the upper dentition and 1.90 mm for the lower dentition.

**Table 1:** Distances of the upper jaw between protocols A and B: mean absolute distance, standard deviation, 95th percentile, and the percentages of the matched distances that are larger than 1 or 1.5 mm

Patient number	Mean absolute distance	Standard deviation	95th percentile	% ≥ 1 mm	% ≥ 1.5 mm
1	0.23	0.44	0.54	0.0	0.0
2	0.23	0.25	0.67	0.0	0.0
3	0.35	0.69	0.64	2.1	1.2
4	0.20	0.24	0.55	1.4	0.4
5	0.32	0.34	0.70	0.0	0.0
6	0.43	0.87	0.74	0.5	0.3
7	0.26	0.49	0.71	0.0	0.0
8	0.53	0.45	1.21	11.1	2.4
9	0.38	0.21	0.80	0.0	0.0
10	0.17	0.28	0.46	2.3	1.1
11	0.51	0.76	1.24	10.2	2.2
12	0.20	0.22	0.47	0.0	0.0
13	0.32	0.48	0.74	0.0	0.0
14	0.46	0.78	0.85	0.0	0.0
15	0.38	0.23	0.73	0.0	0.0
16	0.44	0.26	0.90	0.0	0.0
17	0.94	0.59	1.86	45.5	20.7
18	0.33	0.53	0.83	2.2	0.6
19	0.67	0.54	1.67	27.4	8.0
20	0.38	0.64	1.36	8.9	3.8
Mean	0.39	0.46			
SE	0.04	0.05

**Table 2:** Distances of the lower jaw between protocols A and B: mean absolute distance, standard deviation, 95th percentile, and the percentages of the matched distances that are larger than 1 or 1.5 mm

Patient number	Mean absolute distance	Standard deviation	95th percentile	% ≥ 1 mm	% ≥ 1.5 mm
1	0.33	0.66	0.83	0.0	0.0
2	0.14	0.25	0.39	0.0	0.0
3	0.47	0.37	1.23	10.2	1.4
4	0.30	0.40	1.05	5.3	2.6
5	0.20	0.36	0.44	0.0	0.0
6	0.19	0.33	0.66	3.2	1.8
7	0.26	0.43	0.56	0.0	0.0
8	0.32	0.31	0.80	2.3	1.1
9	0.21	0.25	0.51	0.0	0.0
10	0.22	0.42	0.74	3.5	1.5
11	0.31	0.42	1.18	6.8	2.6
12	0.17	0.18	0.43	0.0	0.0
13	0.14	0.26	0.30	0.0	0.0
14	0.29	0.31	0.69	0.0	0.0
15	0.20	0.30	0.55	0.0	0.0
16	0.28	0.48	0.75	0.0	0.0
17	0.86	1.37	1.90	39.2	21.7
18	0.30	0.42	1.13	5.9	2.6
19	0.66	0.48	1.60	22.3	6.3
20	0.18	0.32	0.50	1.5	0.6
Mean	0.30	0.42			
SE	0.04	0.06			

## Discussion

Until now, CBCT scans are still subject to artifacts at the level of the dentition. These artifacts are not only caused by brackets and restorations, but, to a lesser extent, the dental enamel also causes these artifacts [21–23], the so-called beam hardening effect.

When matching a digital model to the dentition of the CBCT, the complete digital model disappears in the dentition of the CBCT. There are two possible explanations for this phenomenon. First, the digital model is smaller than the real dentition; however, several studies on the validity of digital models have shown that digital models are accurate and provide a real representation of the dentition [23–25]. Second, the dentition in the CBCT scan is larger than the real dentition. When considering the beam hardening effect, this will result in a larger representation of the teeth in a CBCT scan. Consequently, for proper surgical planning and fabrication of an intermediate splint, the representation of the dentition based on the CBCT is not reliable. Therefore, a digital dental model must be integrated into the CBCT scan. For this purpose, we suggest a faster method with less radiation exposure for the patient than the Swennen method [3] used at many surgical centers.

We summarized the data for all patients in Tables 1 and 2. Patient 17 had the poorest results. A closer look at the matched models for this patient shows that the impression in step 3A was not replaced correctly in the mouth as it was in step 1A. The impressions from protocol B (Rangel) were matched correctly on the markers and had a proper fit to the CBCT dentition. This means that the poor results were caused by the incorrect replacement of the impression in protocol A (Swennen) and are not caused by an inconsistency in protocol B (Rangel).

Even though patient 17 has these poor results, the mean distance for all patients for the upper jaw is 0.39 mm and for the lower jaw is 0.30 mm. This is comparable to the study of De Waard et al. 2016 [26], who found mean distances of 0.30 and 0.27 mm for the upper and lower jaw, respectively.

In 3D virtual planning, the time needed to perform the digital planning is important. If virtual surgery planning takes too long, the costs will not outweigh the benefits. Swennen [3] reported that total computational time is about 50 min. Yang et al. [18] developed an integration method using a splint with fiducial markers. They reported that it takes about 60 min to fabricate the intra-oral template with the fiducial markers. Unfortunately, they do not describe how long the data handling process took, but with three superimposition procedures, this should take at least 15 min. In our study, the time to place the markers took about 10 min and data processing afterwards took about 15 min. Therefore, our new method is fast and cost-effective.

None of our patients reported any discomfort due to placement of the markers. The tissue adhesive that was used in this study consisted of N-butyl 2-cyanoacrylate and was approved for clinical use in early 1996. Since then, it has been widely used for closure of superficial lacerations under low tension in a variety of different surgeries [27, 28].

The use of AlgiNot™ in the Swennen protocol has a few flaws. AlgiNot™ is a single-vinyl A-silicone that comes in self-mixing cartridges. An AlgiNot™ impression produces a higher image quality of the dentition than an impression made with alginate, which may give a better representation of the dentition in the virtual model. However, it has been shown that digital models made from alginate impressions are accurate and reliable [25, 29, 30]. Additionally, in conventional orthognathic planning, alginate impressions are always used to make the plaster models. Intermediate splints made on those models have been successfully used during surgeries for decades, so it is questionable how much influence the use of AlgiNot™ has on the clinical outcome. AlgiNot™ has the disadvantage that the material is rather stiff and rigid, and this complicates the repositioning of the AlgiNot™ impression tray in the mouth (step 3A of protocol A). Sometimes the brackets and orthodontic wires hamper the proper seating of the impression. As we have seen with patient 17 in our study, this can result in incorrect repositioning of the impression tray, which results in an incorrect representation of the patient’s dental arches in the virtual model. This could cause incorrect virtual treatment planning and an imperfect fit of the intermediate splints during the operation. In the new protocol, repositioning of the impressions in the mouth is not needed, which will result in a more precise virtual model.

This method has some limitations. The use of N-butyl 2-cyanocrylate and the matching procedure have already been addressed in Rangel et al. 2012 [8]. Furthermore, in the present study, both protocols A (Swennen) and B (Rangel) were only tested on one CBCT machine. It is unknown if using a different CBCT machine would give a different matching result. This should be tested in the future.

The use of markers is also a limitation of this study. During the CBCT scanning, the markers can come lose, when the patient is touching the markers with his tongue. This is not a major issue, as we could get a good registration of the dental surfaces with three markers. In none of the patients, any of the markers came lose during the CBCT scanning, so the matchings could be performed with five markers in all patients. The same problem occurs when the markers do not remain embedded in the impression. In this case, a clear markerspot is visible in the impression, where the marker can be repositioned properly. And the same as with the CBCT, a good registration can be achieved with three markers. In three patients, we had to reposition the markers into their spot, which was clearly visible in the impression.

Another limitation is that protocol B (Rangel) is only tested on impressions and not yet with an intra-oral scanner. We think that this new method will be especially useful, when intra-oral scanners for imaging the dentition are more commonly used. Most other protocols [3, 9, 10, 15–18, 31] use extra tools that cannot be used with intra-oral scanners, since they need an impression of the dentition. In our protocol, the practitioner can scan the markers with an intra-oral scanner. The voxel values can be digitally added to the intra-oral scan, and matching protocol B can be used to integrate the model. Using an intra-oral scanner will probably provide a more consistent result because no markers will be lost during any of the steps in the protocol.

## Conclusion

This study shows that the new protocol B (Rangel) for integrating digital dental models into CBCT scans is clinically accurate, faster, exposes the patient to less radiation (for protocol B only one CBCT scan is needed), and has a lower risk of positional errors of the impressions than protocol A (Swennen). Moreover, the protocol can also be applied when intra-oral scans of the dentition are made instead of alginate impressions.
